# Poisonous Caterpillar-Inspired Chitosan Nanofiber Enabling Dual Photothermal and Photodynamic Tumor Ablation

**DOI:** 10.3390/pharmaceutics11060258

**Published:** 2019-06-02

**Authors:** Hyeong Sup Yu, Hongsuk Park, Thang Hong Tran, Sung Yeon Hwang, Kun Na, Eun Seong Lee, Kyung Taek Oh, Dongyeop X. Oh, Jeyoung Park

**Affiliations:** 1 Department of Biotechnology, The Catholic University of Korea, 43 Jibong-ro, Bucheon-si, Gyeonggi-do 14662, Korea; skleelsk@naver.com (H.S.Y.); kna6997@catholic.ac.kr (K.N.); 2 Division of Endocrinology, Metabolism & Lipid Research, Washington University School of Medicine, Saint Louis, MO 63110, USA; hongsuk.park@wustl.edu; 3 Research Center for Bio-Based Chemistry, Korea Research Institute of Chemical Technology (KRICT), Ulsan 44429, Korea; thangth@krict.re.kr (T.H.T.); crew75@krict.re.kr (S.Y.H.); 4 Advanced Materials and Chemical Engineering, University of Science and Technology (UCT), Daejeon 34113, Korea; 5 College of Pharmacy, Chung-Ang University, 84 Heukseok-ro, Dongjak-gu, Seoul 06974, Korea

**Keywords:** pH-sensitive chitosan nanofiber, photothermal gold nanoparticles, photodynamic chlorin e6, synergistic tumor therapy, tumor-targeted drug delivery system

## Abstract

As caterpillars detect the presence of predators and secrete poison, herein, we show an innovative and highly effective cancer therapeutic system using biocompatible chitosan nanofiber (CNf) installed with a pH-responsive motif that senses tumor extracellular pH, pH_e_, prior to delivering dual-modal light-activatable materials for tumor reduction. The filamentous nanostructure of CNf is dynamic during cell interaction and durable in blood circulation. Due to its amine group, CNf uptakes a large amount of photothermal gold nanoparticles (AuNPs, >25 wt %) and photodynamic chlorin e6 (Ce6, >5 wt %). As the innovative CNf approaches tumors, cationic CNf effectively discharges AuNPs connected to the pH-responsive motif via electrostatic repulsion and selectively binds to tumor cells that are generally anionic, via the electrostatic attraction accompanied by CNf. We demonstrated via these actions that the endocytosed Ce6 (on CNf) and AuNPs (free from CNf) significantly elicited tumor cell death under light irradiation. As a result, the synergistic interplay of thermogenesis and photodynamic action was observed to switch on at the pH_e_, resulting in a striking reduction in tumor formation and growth rate upon light exposure.

## 1. Introduction

The tumor and its key issues remain a burden on human health and the health care system. Although current clinical approaches, such as surgery, chemotherapy, and radiotherapy, have been applied for therapeutic tumor removal or to preclude tumor development, these treatments are associated with many side effects, complications, and drug resistance. To minimize such traumas, several drug delivery systems (DDSs) have been suggested as anti-tumor agents for use as internal medicine methodologies. However, studies are ongoing in the quest for more effective, astute, rapid, and safer next-generation DDS tools for tumor therapy [1,2,3,4,5,6,7].

The anti-predatory behavior of caterpillars functions as an approach for next-generation DDS. Poisonous caterpillars have evolved their adaptive strategies owing to their constant struggle against predators such as spiders. Poison is used for primary defense under specific environmental conditions such as the detection of predatory behavior. Similarly, an ideal DDS of an antitumor drug should be effectively released from the carrier when a tumor-specific environment is perceived. To understand a caterpillar-mimetic DDS, the following two criteria for material design were established: (1) an effective tumor-targeted drug (poison) and (2) an innovative environmental-recognition and worm-like structure to function as the drug carrier (predator recognition) [1,2,3,4,5,6,7].

As an anti-tumor weapon, we have selected dual-modal phototherapy where thermogenesis-based photothermal therapy (PTT) is combined with reactive oxygen species (ROS)-based photodynamic therapy (PDT). PTT employs the conversion of light to heat energy to elicit hyperthermia in tumor regions using gold nanoparticles (AuNPs) or graphene oxide. PDT, on the other hand, relies on ROS production when a photosensitizer such as chlorin e6 (Ce6) reacts with oxygen present in tissues to kill tumor cells during irradiation [1,2,3,4,5,6,7]. Since sole-modal therapy has failed to manage the rising rates in various types of diseases, combinatorial treatments have been widely considered and have garnered a significant amount of interest [8,9,10,11,12]. Agreeing with this trend, there is a growing appreciation for the integration of PTT and PDT to function as a highly effective approach in tumor control [13,14,15].

As an innovative vehicle for photosensitizers, we have designed a biocompatible and pH-sensitive worm-like nanofiber. Chitosan nanofibers (CNfs) are a promising backbone for drug vehicles based on the following three rationales: (1) CNf is biomass-derived and biodegradable [16,17,18,19,20]. CNf is chemically modifiable for drug loading owing to its abundance in amine groups and its association with a relatively low immune reaction and disease transfer possibility, unlike animal-derived collagen fibers. (2) CNf is positively charged and thus can serve as a tumor extracellular pH (pH_e_)-responsive carrier. If CNf was employed to carry pH-sensitive proton-accepting payloads, a safe transport of the payloads to the tumor site at physiological pH (7.4) would occur. However, sudden destabilization or disintegration at pH_e_ (pH 6.8) would also result, owing to the repulsive forces of the positive charges when there is an efficient release of payloads near the tumor [21,22,23,24,25]. Furthermore, CNf covalently conjugated to a drug can strongly bind to the anionic tumor. (3) CNf has a worm-like fiber structure. It has been noted that the shape of DDS tools can affect the journey of particles after entering the body. Filamentous nanostructures have durable circulation in blood and efficient cellular internalization via the adsorptive endocytic pathway [26,27]. CNf may therefore have temporal and spatial advantages as a potent delivery vehicle for tumor-targeted DDS.

**Figure 1:**
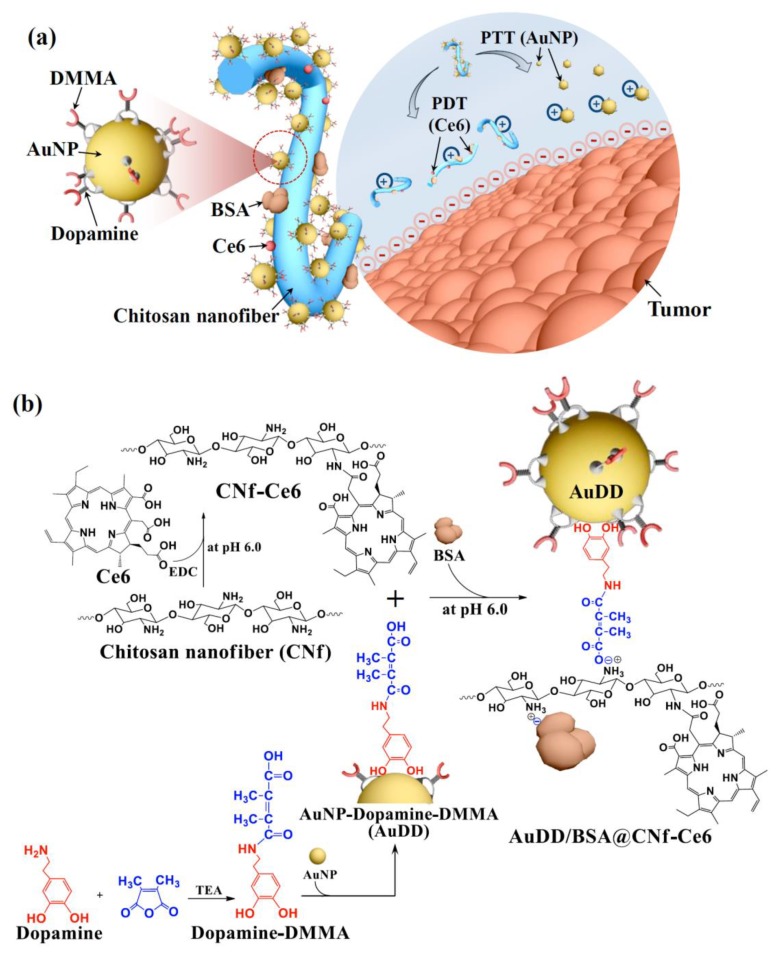
(**a**) Schematic concept for the integration of pH_e_-sensitive chitosan nanofiber with AuNP, Ce6, and BSA. (**b**) Scheme for the synthesis of AuDD/BSA@CNf-Ce6.

The objective of this study was to develop pH_e_-sensitive filamentous chitosan-based nanomaterial for synergistic tumor therapy via the combination of PTT and PDT. Beyond their role as building blocks, we also included a variety of ingredients on the chitosan backbone to serve as decorations (Figure 1a).

pH_e_-sensitive dopamine-2,3-dimethylmaleic acid (DMMA) [22,23] was coated on a heat-producible AuNP [28,29] to synthesize AuNP-dopamine-DMMA (AuDD). A photosensitizer, Ce6 [5], was covalently attached to the body of the chitosan nanofiber to synthesize CNf-Ce6. We also electrostatically linked negatively charged bovine serum albumin (BSA) to the cationic CNf-Ce6 to improve its stability as a drug delivery carrier during blood circulation [30]. In a neutral pH environment, AuDD is linked to the chitosan backbone owing to its electrostatic interaction (Figure 1b). However, AuDD is removable from the cationic chitosan backbone at acidic pH_e_, an action mainly ascribed to the change in charge of DMMA. Hence, based on the unique pH_e_-involved strategy, our DDS agent (CNf), which was covalently grafted to Ce6 and electrostatically coupled to AuDD and BSA (AuDD/BSA@CNf-Ce6), was designed to be pH_e_-sensitively transformed to release individual antitumor payloads of Ce6 and AuDD. The separated CNf-Ce6 and AuDD would also react with the negatively charged tumor cell surface, enhancing their uptake into the tumor cells. The notion of tumor degeneration via biocompatible CNf-based phototherapy with pH_e_ selectivity would serve as a novel and fundamental development for next-generation DDS. This pH_e_-sensitive CNf may be more significant than any known nanofiber systems that have been developed to date.

## 2. Materials and Methods

### 2.1. Materials

The α-chitin powder from shrimp shells, sodium hydroxide (NaOH), dopamine hydrochloride, 2,3-dimethylmaleic anhydride, *N*, *N*′-dimethylformamide (DMF), trimethylamine (TEA), pyridine, anhydrous diethyl ether, gold (III) chloride hydrate (HAuCl_4_), sodium citrate, *N*-(3-dimethylaminopropyl)-*N*′-ethylcarbodiimide hydrochloride (EDC), sodium tetraborate, bovine serum albumin (BSA), 9,10-dimethylanthracene, formaldehyde, and 4′,6-diamidino-2-phenylindole dihydrochloride (DAPI) were purchased from Sigma-Aldrich (St. Louis, MO, USA). Hydrochloric acid (HCl) was bought from Daejung (Seoul, Korea). Ce6 was acquired from Frontier Scientific Inc. (Logan, UT, USA). The BCA protein assay kit was bought from Thermo Fisher Scientific Inc. (Walthan, MA, USA). Dulbecco’s modified Eagle’s medium (DMEM), Roswell Park Memorial Institute (RPMI) 1640 medium, phosphate-buffered saline (PBS), ethylenediaminetetraacetic acid (EDTA), fetal bovine serum (FBS), penicillin, and streptomycin were purchased from Welgene Inc. (Seoul, Korea). Wheat Germ Agglutinin-Alexa Fluor^®^ 488 conjugate (WGA-Alexa Fluor^®^ 488) was purchased from Life Technologies (Carlsbad, CA, USA). The Cell Counting Kit-8 (CCK-8) was obtained from Dojindo Molecular Technologies Inc. (Santa Clara, CA, USA). FITC annexin V apoptosis detection kit I was acquired from BD Pharmingen (San Diego, CA, USA).

### 2.2. Preparation of Chitosan Nanofiber (CNf)

The α-chitin powder (5 g) was immersed in a 30 wt % NaOH aqueous solution (125 mL). The suspension was then heated at 80 °C for 4 h in a nitrogen atmosphere followed by centrifugation at 10,000 rpm for 10 min at 5 °C. After the removal of the supernatant, the pellet was re-constituted in deionized (DI) water (125 mL). The base dilution processes were repeated three times. The suspension was then dialyzed with DI water until a pH of 7 was obtained. The concentration of the suspension was adjusted to 1 wt % by adding DI water. The pH of the suspension was adjusted to 4 by adding several drops of acetic acid, followed by homogenization using a high-performance grinder (MKCA6-3; Masuko Sangyo Co., Ltd., Kawaguchi, Japan) with a rotation speed of 1500 rpm. The grinder treatment was performed with a clearance gauge of −1.5 (corresponding to a 0.15 mm shift) from the zero position. After nanofibrillization, the purified suspension was ultrasonicated for 10 min (amplitude, 50%; pulse on, 10 s; and pulse off, 5 s) by a 750 W probe ultrasonic processor (Sonics, Vibra cell, Sonic & Materials, Inc., Newtown, CT, USA). The aqueous CNf suspension or its freeze-dried form was stored at 4 °C [31]. The degree of deacetylation of the CNf material was confirmed using a Nicolet iS50 FT-IR spectrometer (Thermo Fisher Scientific, Walthan, MA, USA) and via titrating the amine groups on the surface of CNf [32,33].

### 2.3. Synthesis of AuNP-Dopamine-DMMA (AuDD)

To prepare AuDD, we first reacted dopamine (1 g) with 2,3-dimethylmaleic anhydride (1.7 g) in DMF (15 mL) containing TEA (1 mL) and pyridine (1 mL) at 25 °C for 3 d to produce dopamine-DMMA. The solution was recrystallized using excessive anhydrous diethyl ether and the precipitate lyophilized. The yield of dopamine-DMMA was 72.3 ± 4.8 wt % and the chemical structure was analyzed using a Bruker 300 MHz NMR Spectrometer (Bruker, Ettlingen, Germany). Next, to prepare intact AuNP, HAuCl_4_ (2.5 mmoL) in 0.4 M NaOH (19.4 mL) was heated to 110 °C (for 30 min) and then 85 °C (for 10 min). The obtained AuNP solution was mixed with sodium citrate (0.1 mol; 0.6 mL) for 10 min. The resulting solution was ultracentrifuged at 25,000 rpm for 10 min at 5 °C to separate the particles from the non-reacted chemicals. The dried precipitates (0.1 g) were sonicated in DI water for dispersal of the AuNP in the DI water [33]. Dopamine-DMMA (1.5 g) was then mixed with the AuNP (30 mg) dispersed in DI water (45 mL) and 5 mM sodium tetraborate (20 mL) for 1 d to prepare AuDD. The concentration of dopamine-DMMA bound to the AuNP was calculated after measuring the weight of the dried precipitate (AuDD), following ultracentrifugation of the solution at 25,000 rpm for 10 min at 5 °C. The yield of AuDD was 71.4 ± 4.2 wt % and was calculated after lyophilization.

### 2.4. Preparation of CNf-Based Nanostructures

To prepare CNf-Ce6, CNf (200 mg) was dissolved in 50 mM HCl (50 mL) using a sonicator (60 Hz for 30 min) at 25 °C. Ce6 (10 mg) was dissolved in 50 mM NaOH (20 mL) containing EDC (5 mg) and mixed with the CNf solution for 1 d. The pH of the mixed solution was adjusted to 6.0 by the gradual addition of 50 mM NaOH. The solution was then dialyzed using a dialysis membrane tube (Spectra/Por^®^ MWCO 1 kDa) against DI water for 3 d to remove the non-reacted chemicals, prior to lyophilization. The production yield of CNf-Ce6 was 80.4 ± 3.1 wt %. The amount of Ce6 conjugated to CNf was determined after analyzing the Ce6 fluorescent intensity in the CNf-Ce6 solution at λ_ex_ (450 nm) and λ_em_ (670 nm) using a fluorescence RF-5301PC spectrofluorometer (Shimadzu, Japan).

We then prepared different CNf-Ce6-based nanostructures using AuDD and BSA. CNf-Ce6 (75 mg) was dispersed in 50 mM HCl (50 mL) using a sonicator (60 Hz for 30 min) at 25 °C. BSA (100 mg) was dissolved in 50 mM NaOH (20 mL) and mixed with the CNf-Ce6 solution for 8 h [34]. The pH of the mixed solution was adjusted to 7.4 by the slow addition of 50 mM NaOH. The resulting precipitation of BSA@CNf-Ce6 was then lyophilized. The production yield of BSA@CNf-Ce6 was 81.5 ± 6.1 wt %. The amount of BSA attached to CNf-Ce6 was calculated using a BCA protein assay kit. In addition, the dispersed CNf-Ce6 (15 mg) or BSA@CNf-Ce6 (15 mg) in 5 mM sodium tetraborate solutions (20 mL) was mixed with AuDD (75 mg) at 25 °C for 8 h to generate AuDD@CNf-Ce6 or AuDD/BSA@CNf-Ce6. The resulting solutions were ultracentrifuged at 25,000 rpm for 10 min at 5 °C to separate the fibers from the nonreacted chemicals. The yields of AuDD@CNf-Ce6 and AuDD/BSA@CNf-Ce6 were 70.2 ± 4.3 wt % and 73.5 ± 2.9 wt %, respectively and were calculated after lyophilization. The AuDD concentrations in AuDD@CNf-Ce6 and AuDD/BSA@CNf-Ce6 were analyzed using ICP-MS (Thermo Scientific Inc., Walthan, MA, USA).

### 2.5. Characterization of AuDD and CNf-Based Samples

A Zetasizer 3000 instrument (Malvern Instruments, Westborough, PA, USA) was used to measure the particle size of AuDD in PBS (pH 7.4, 150 mM). The morphology of the CNf-based samples or AuDD (0.1 mg/mL, 150 mM PBS pH 7.4) was analyzed using a TEM (JEM 1010, JEOL, Peabody, MA, USA). The light absorbance of CNf-based samples or AuDD dispersed in PBS (pH 7.4) was observed using a Cary 1E UV/visible spectrophotometer (Varian Inc., Palo Alto, CA, USA). Visualization of the CNf-based samples or AuDD at pH 7.4 or 6.8 was monitored using a Nikon microscope equipped with a visible and NIR hyperspectral camera (CytoViva, Auburn, AL, USA). The zeta-potential change in CNf-based samples (0.1 mg/mL) at pH 7.4 or 6.8 was measured using Zetasizer 3000. Prior to measuring, the CNf-based samples were stabilized at 25 °C for 2 h. In addition, when the PBS (150 mM, pH 7.4 or 6.8) containing CNf-based samples or AuDD was irradiated using an 808 nm laser source (2 W/cm^2^ for 5 min), the temperature change in solution was monitored using a probe-type thermometer (905-T1, Testo Inc., West Chester, PA, USA) and a thermographic camera (T335, FLIR Systems Inc., Seoul, Korea) [33].

### 2.6. NIR Fluorescence Analysis

For quantitative analysis, Ce6 fluorescence intensity change in the CNf-based samples (equivalent to Ce6 10 µg/mL) or free Ce6 (10 µg/mL) at pH 7.4 and 6.8 was measured at λ_ex_ (400 nm) and λ_em_ (600–750 nm). The NIR fluorescence images from wells containing the CNf samples (equivalent to Ce6 10 µg/mL) or free Ce6 (10 µg/mL) at pH 7.4 and 6.8 were visualized using a Kodak image station (λ_ex_, 635 nm; λ_em_, 720 nm). The generation of singlet oxygen from the CNf-based samples or free Ce6 was measured using a fluorescence spectrofluorometer by analyzing the fluorescence of 9,10-dimethylanthracene (λ_ex_, 360 nm; λ_em_, 380–550 nm) [35,36]. Briefly, the CNf samples (equivalent to Ce6 10 µg/mL) or free Ce6 (10 µg/mL) in PBS (pH 7.4 and 6.8, 150 mM) were mixed with 9,10-dimethylanthracene (20 mM). The resulting solution was then irradiated using a 670 nm laser source (5.2 mW/cm^2^ for 10 min). After 1 h incubation, the fluorescence change of 9,10-dimethylanthracene (F_f_–F_s_), calculated by subtracting the fluorescence of CNf samples (F_s_) from the fluorescence of pure 9,1-dimethylanthracene (F_f_), was plotted. The fluorescence of 9,10-dimethylanthracene decreases because of its selective capture of singlet oxygen [35].

### 2.7. In Vitro Cellular Uptake Study

Human breast carcinoma MDA-MB-231 cells (obtained from the Korean Cell Line Bank) were cultured in RPMI-1640 medium containing 10% FBS and 1% penicillin-streptomycin at 37 °C and 5% CO_2_. The cells were incubated with the CNf-based samples (200 μg/mL, equivalent to Ce6 10 μg/mL) or AuDD (50 μg/mL) at pH 7.4 and 6.8 (incubation time, 8 h). The cellular uptake value [number (N) of AuDDs taken up per cell] for the AuDD samples was acquired using ICP-MS [33]. For flow cytometry analysis, the cells were incubated with the CNf-based sample (equivalent to Ce6 10 µg/mL), free Ce6 (10 µg/mL), or Ce6 dye-tagged AuNP (equivalent to Ce6 10 µg/mL) at pH 7.4 and 6.8 (incubation time: 8 h). Following incubation, the cells were washed three times with fresh 150 mM PBS (pH 7.4) and analyzed using a FACSCalibur™ flow cytometer (Becton Dickinson, USA). Visualization of the cells incubated with AuDD/BSA@CNf-Ce6 sample (200 μg/mL) at pH 7.4 and 6.8 for 8 h were performed using a Nikon microscope. Next, the cells incubated with each sample (equivalent to Ce6 10 μg/mL) at pH 7.4 and 6.8 for 8 h were stained with DAPI (nuclei stain) and WGA-Alexa Fluor^®^ 488 (cell membrane staining) for cell analysis using a confocal microscope (Meta LSM710, CarlZeiss, Oberkochen, Germany).

### 2.8. In Vitro Cell Viability

The phototoxicity of the tumor cells was examined under light irradiation using a 670 or 808 nm laser source. Cells were incubated with CNf-based samples (200 μg/mL, equivalent to Ce6 10 μg/mL), free Ce6 (10 μg/mL), or AuDD (50 μg/mL) at pH 7.4 and 6.8 for 8 h followed by three washings using fresh PBS (150 mM, pH 7.4). The cells were then irradiated at a light intensity of 5.2 mW/cm^2^ using a 670 nm laser source for 10 min (PDT) and/or at a light intensity of 2 W/cm^2^ using an 808 nm laser source for 5 min (PTT), or repeatedly [5,36]. Cell viability was measured using a CCK-8 assay. In addition, the basal cytotoxicity of the CNf-based samples or AuDD was evaluated after a 24 h treatment without light irradiation. To further evaluate cell apoptosis induced by the photothermal/photodynamic treatment of the AuDD/BSA@CNf-Ce6 sample, the Annexin-V-FITC/PI staining method was performed [37]. MDA-MB-231 cells were first seeded into a 12-well plate at a density of 1 × 10^4^ cells per well at 37 °C in a 5% CO_2_ atmosphere for 24 h. The cells were then washed three times with fresh PBS (150 mM, pH 7.4) to remove the dead cells and were incubated with the AuDD/BSA@CNf-Ce6 sample (200 μg/mL, equivalent to Ce6 10 μg/mL) dispersed in culture medium (pH 7.4 and 6.8) at 37 °C for 8 h. The cells were then washed three times with fresh PBS (150 mM, pH 7.4) to remove the AuDD/BSA@CNf-Ce6 samples that were not uptaken, followed by exposure to a 670 nm laser (5.2 mW/cm^2^) for 10 min (PDT irradiation) and/or an 808 nm laser (2 W/cm^2^) for 5 min (PTT irradiation). After laser irradiation, the cells were incubated with fresh culture medium at 37 °C for 24 h. Following incubation, the cells were collected and resuspended in 500 μL of binding buffer, and Annexin V-FITC and PI added, according to the manufacturer’s recommendation. The treated cells were incubated in the dark for 15 min at 25 °C and then analyzed using FACSCalibur™ flow cytometry.

### 2.9. Animal Care

All animal experiments were conducted using 6- to 8-week-old female nude mice (BALB/c, nu/nu mice, Institute of Medical Science, Japan). The nude mice were maintained using the guidelines (animal ethics number: 2018-016, approved by the Catholic University of Korea) approved by the Institutional Animal Care and Use Committee (IACUC) of the Catholic University of Korea (Republic of Korea).

### 2.10. In Vivo Uptake Test

For the in vivo experiments, female nude mice were inoculated with MDA-MB-231 tumor cells via subcutaneous injection of 1 × 10^7^ cells suspended in 150 mM PBS (pH 7.4). When tumor volume reached 100 mm^3^, each CNf-based sample (50 mg/kg, equivalent to Ce6 2.5 mg/kg) or free Ce6 (2.5 mg/kg) was intravenously injected into the MDA-MB-231 tumor-bearing nude mice through the tail vein. Live fluorescent images of the MDA-MB-231 tumor-bearing nude mice were obtained using an Image Station 4000 MM (Kodak, Rochester, NY, USA). Total photon counts per centimeter squared per steradian (p/s/cm^2^/sr) in the organs were measured using the Image Station 4000 MM. At 8 h post-injection, the nude mice were sacrificed, and the excised tumor and organs (brain, lung, heart, liver, kidney, and spleen) analyzed for the biodistribution study.

### 2.11. In Vivo Micro-CT Scan

The micro-CT imaging of MDA-MB-231 tumor-bearing nude mice that were intravenously injected with AuDD/BSA@CNf-Ce6 (50 mg/kg) were obtained at 8 h post-injection using a micro-CT imaging scanner (CLS140083, PerkinElmer Inc., Waltham, MA, USA) under appropriate anesthesia.

### 2.12. In Vivo Thermal Imaging

MDA-MB-231 tumor-bearing nude mice that were intravenously injected with the CNf-based sample (50 mg/kg), AuDD (12.5 mg/kg), or saline (control) were locally irradiated (8 h post-injection) at a light intensity of 2 W/cm^2^ using an 808 nm laser source for 5 min (PTT). The thermal body images of mice were captured using a thermal imaging camera [33].

### 2.13. Tumor Growth Measurement

MDA-MB-231 tumor-bearing nude mice intravenously injected with the CNf-based sample (50 mg/kg, equivalent to Ce6 2.5 mg/kg), free Ce6 (2.5 mg/kg), AuDD (12.5 mg/kg), or saline (control), were locally irradiated (after 8 h injection) at a light intensity of 5.2 mW/cm^2^ using a 670 nm NIR laser for 40 min (PDT) and subsequently at a light intensity of 2 W/cm^2^ using an 808 nm NIR laser for 5 min (PTT). Tumor volume of nude mice was determined using the following formula: tumor volume = length × (width)^2^/2. In addition, the relative tumor volume change (V_t_/V_0_) was calculated from the ratio of V_t_ (the tumor volume at a given time) to V_0_ (the initial tumor volume). Change in body weight of mice (W_t_/W_0_) was calculated from the ratio of W_t_ (the body weight at a given time) to W_0_ (the initial body weight) [33,35].

### 2.14. Statistics

All results were analyzed via a Student’s t-test or analysis of variance (ANOVA) with *p* < 0.01 (**) as the significance level. The MINITAB^®^ release 14 statistical software program was used for all statistical analyses.

## 3. Results and Discussion

### 3.1. Synthesis and Characterization of AuDD/BSA@CNf-Ce6

As shown in the schematic illustration (Figure 1b), stepwise synthesis was adopted to prepare the pH_e_-responsive AuDD/BSA@CNf-Ce6, the backbone of which was obtained by facile deacetylation and ultrasonication of shrimp shell-originated α-chitin. The degree of deacetylation was 44.0% as determined by Fourier transform infrared spectroscopy (Table S1) [31,32]. When measured via the titration method, the degree of deacetylation was 26.9 ± 3.3%; this lower value is because of the inaccessibility of protons to the CNf core [38]. Additionally, the degree of DMMA attached to dopamine (defined as the number of DMMA molecules per dopamine molecules) was estimated as 1.7, based on calculations using ^1^H-NMR peak values at δ 1.6 ppm (–C*H*_3_ from DMMA) and δ 2.8 ppm (–C*H*_2_– from dopamine) (Figure S1). Dopamine-DMMA was coated on the surface of the gold nanoparticle (AuNP) via catecholic coordination [39,40], resulting in AuDD formation. We obtained a size that was approximately 24.4 nm in diameter, along with a spherical shape for the AuDD (Figure S2). To achieve the optimal feeding ratio of AuDD conjugation to the CNf body, we combined 2-, 5-, and 10-fold AuDD with CNf; the coupled ratios were 7% (AuDD_7_@CNf), 24% (AuDD_24_@CNf), and 40% (AuDD_40_@CNf), respectively. The hyperspectral imaging analysis demonstrated that AuDDs in red were electrostatically associated with CNfs in yellow, under the physiological pH condition (Figure S3). It is noteworthy that AuDD_40_@CNf resulted in excessive conjugation of AuDD to the CNf backbone, which led to particle aggregation. The optimal feeding ratio with the 5-fold AuDD reactant freed AuDD_24_@CNf to accommodate the non-aggregate nanoparticle; this allowed further decoration of BSA and Ce6 to synthesize AuDD/BSA@CNf-Ce6 (Figure S4). We then prepared three types of CNf-grafted Ce6 (BSA@CNf-Ce6, AuDD@CNf-Ce6, and AuDD/BSA@CNf-Ce6) and calculated the weight fractions of AuDD, Ce6, and BSA in each of the CNf construct (Table 1).

**Figure 2:**
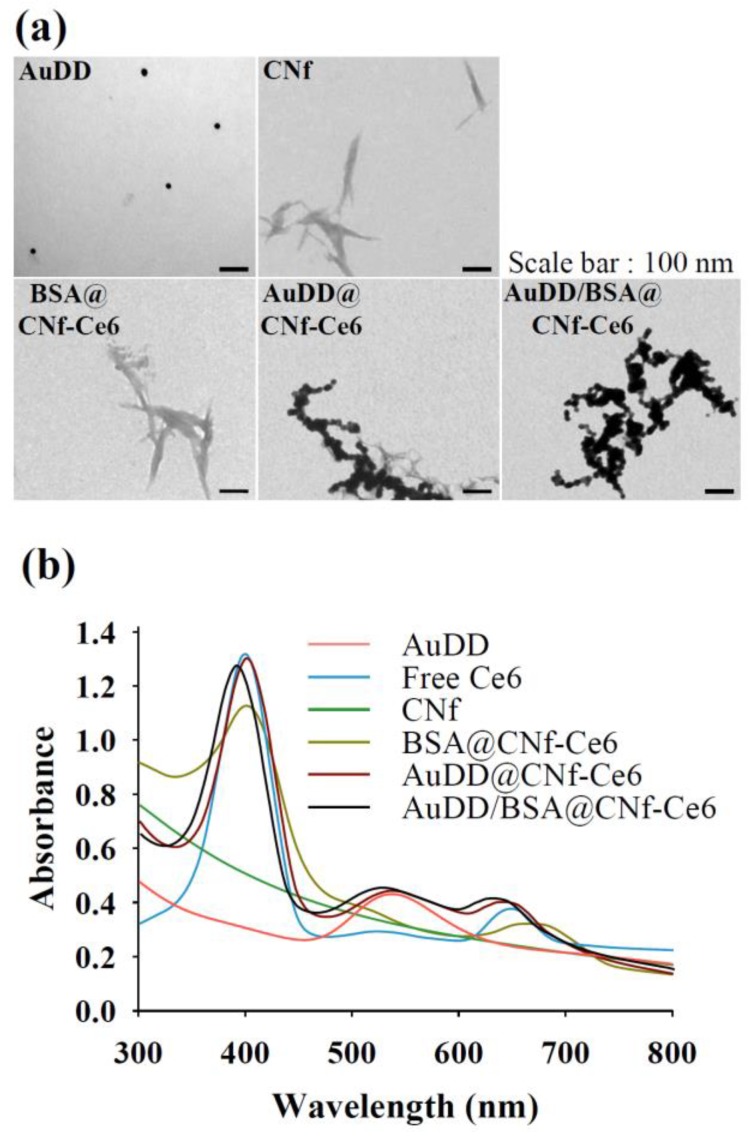
(**a**) TEM images of each nanostructure. (**b**) UV/Vis spectrum of all samples.

The coupled weight portions of AuDD, Ce6, and BSA to CNfs were 24.9–25.5%, 4.9–5.2%, and 3.9–4.1%, respectively. The morphology of each nanostructure was visualized using transmission electron microscopy (TEM), which showed CNfs having widths of 10–30 nm and lengths of 100–550 nm (Figure 2a); the AuDD attached to CNf was observed in AuDD@CNf-Ce6 and AuDD/BSA@CNf-Ce6. UV/Vis identification was conducted to confirm the binding of AuDD and Ce6 (Figure 2b). The individual peaks in AuNP (500–600 nm) [35,40] representing AuDD, AuDD@CNf-Ce6, and AuDD/BSA@CNf-Ce6 were detected in the spectra. In addition, those of Ce6 (approximately 400 and 670 nm) [41] were measured from free Ce6, BSA@CNf-Ce6, AuDD@CNf-Ce6, and AuDD/BSA@CNf-Ce6.

### 3.2. Investigating the pH-Dependent AuDD/BSA@CNf-Ce6 Operation

**Figure 3:**
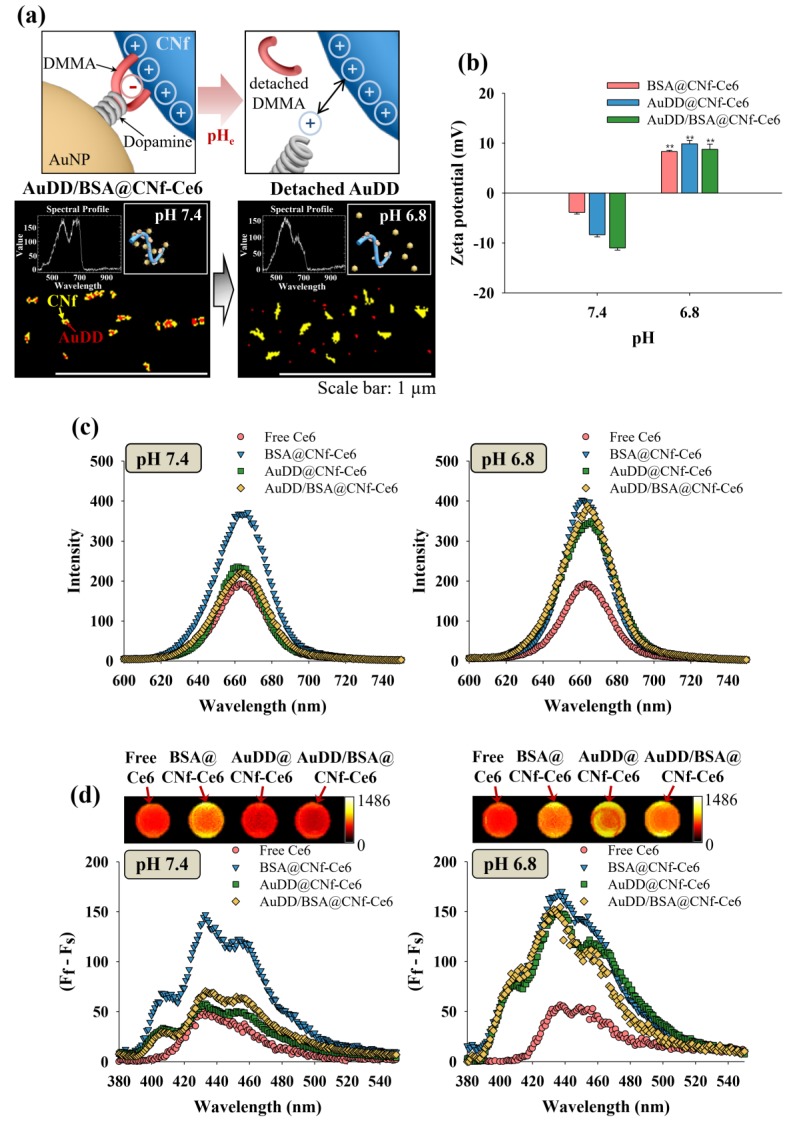
(**a**) Schematic illustration and hyperspectral images of anchored/detached AuDD from the CNf backbone at pH 7.4 and 6.8. (**b**) Changes in zeta potential of CNf-Ce6-based samples at pH 7.4 and 6.8 (mean ± SD, *n* = 3, ** *p* < 0.01 compared to pH 7.4). (**c**) Ce6 emission spectra (λ_ex_ = 400 nm, λ_em_ = 600–750 nm) for the free Ce6 or CNf-Ce6-based samples at pH 7.4 and 6.8. (**d**) 9,10-Dimethylanthracene fluorescence change (F_f_–F_s_) in free Ce6 or CNf-Ce6-based samples at pH 7.4 and 6.8 (lower graphs), and NIR Ce6 fluorescence (λ_ex_ = 635 nm, λ_em_ = 720 nm) images of free Ce6 or CNf-Ce6-based samples at pH 7.4 and 6.8 (upper images). All samples were irradiated for 10 min at a light intensity of 5.2 mW/cm^2^ using a 670 nm laser source.

With increasing evidence that the tumor microenvironment has an acidic extracellular pH, termed pH_e_, we introduced the DMMA-based self-assembly of AuDD/BSA@CNf-Ce6 that safely stores light-activatable materials at physiological pH and discharged them at pH_e_ to inactivate the tumor cells. The characteristic pH_e_ was expected to activate the dynamic disassembly of antitumor photopoisons, photothermal AuNP, and photodynamic CNf-Ce6 (Figure 3a, upper panels). To verify the division of AuDD/BSA@CNf-Ce6 in a pH_e_-dependent manner, we treated the compounds with a pH of 7.4 and 6.8, and detected AuDD in red and CNf-Ce6 in yellow using hyperspectral imaging (Figure 3a, lower panels). Whereas the red AuDD particles were fused with yellow CNf-Ce6 bodies because of their electrostatic interaction under the physiological environment of pH 7.4, the AuDD particles were discharged from the CNf-Ce6 backbones under a tumor environment of pH 6.8. The exposure to pH_e_ resulted in a decoupling of DMMA from AuDD, followed by positively charged dopamine-AuNP formation and its removal from the cationic CNf-Ce6 backbone via electrostatic repulsion [42]. The zeta potential test showed that the surface charges of CNf-Ce6-based materials changed from negative at pH 7.4 to positive at pH 6.8 (Figure 3b), due to the decoupling of DMMA from AuDD at pH 6.8. The three types of CNf-Ce6-based materials, BSA@CNf-Ce6, AuDD@CNf-Ce6, and AuDD/BSA@CNf-Ce6, had negative charges of −3.9, −8.3, and −11.0 mV, respectively, under physiological conditions. All materials displayed positive surface charges around 15.8–17.2 mV at pH_e_, suggesting that the tumor-associated pH (i.e., pH 6.8) can promote electrostatic change in the CNf-Ce6-based nanostructures.

**Figure 4:**
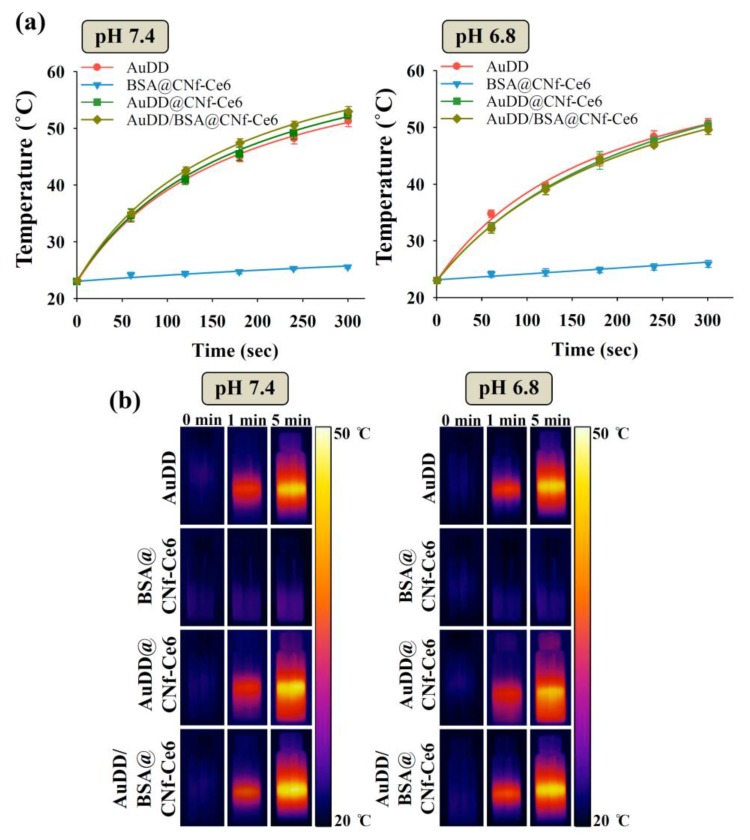
(**a**) Kinetic temperature changes and (**b**) photothermal images of each sample in PBS (150 mM, pH 7.4 and 6.8) irradiated at a light intensity of 2 W/cm^2^ using an 808 nm light source for 5 min (mean ± SD, *n* = 3).

Since AuDD/BSA@CNf-Ce6 contains both photodynamic Ce6 and the photothermal AuNP, we verified the presence of two photoactive chemicals. Figure 3c shows the pH-dependent fluorescent responses of different Ce6-based nanostructures from 640 to 670 nm [43]. Free Ce6 had low fluorescence intensity at both pH 7.4 and 6.8 owing to its hydrophobic interaction-caused auto-quenching [5]. BSA@CNf-Ce6 generated the highest emission peak at pH 7.4 as the fluorescence of Ce6 was de-quenched by BSA. Importantly, while AuDD@CNf-Ce6 and AuDD/BSA@CNf-Ce6 displayed comparable fluorescence intensity to the free Ce6 at pH 7.4, a noticeable increase in their fluorescence emission was observed at pH 6.8. This was a result of the plasmonic effect of AuDD suppressing the fluorescence of AuDD@CNf-Ce6 and AuDD/BSA@CNf-Ce6 under neutral pH conditions [41]. pH_e_, however, activated the dynamic cleavage (by charge–charge repulsion, Figure 3a) and the removal of AuDD from the CNf main body. This means that the removal of AuDD from the CNf main body allows the de-quenched state of Ce6, resulting in an elevated Ce6-generated fluorescence. In addition, near infrared (NIR) images and 9,10-dimethylanthracene fluorescence changes (indicating singlet oxygen generation) [35] for the free Ce6, BSA@CNf-Ce6, AuDD@CNf-Ce6, and AuDD/BSA@CNf-Ce6 demonstrated that AuDD@CNf-Ce6 and AuDD/BSA@CNf-Ce6 pH-dependently released photodynamic Ce6 (Figure 3d). At pH 7.4, AuDD@CNf-Ce6 and AuDD/BSA@CNf-Ce6 were represented by a similar color and low singlet oxygen production when compared to self-quenched free Ce6 under radiation, a result possibly due to the AuDD-induced plasmonic effect on Ce6 [41]. However, a color change to yellow and the singlet oxygen production were dramatically increased at pH 6.8, suggesting that pH_e_ enables AuDD@CNf-Ce6 and AuDD/BSA@CNf-Ce6 to remove AuDD, followed by de-quenching and activation of Ce6. Emerging studies have suggested that the AuNP elicits thermogenesis owing to its strong absorption in the 808 nm region of NIR [28,36,37,44,45,46,47,48,49]. Furthermore, when the AuNP was released from AuDD/BSA@CNf-Ce6 at pH 6.8, we also examined how it has different photothermal effects in response to light (Figure 4).

As shown in Figure 4a, the samples with AuDD rose to a temperature around 50 °C at pH 7.4 and 6.8. In a time-dependent manner, the temperatures of AuDD, AuDD@CNf-Ce6, and AuDD/BSA@CNf-Ce6 increased; however, changes in BSA@CNf-Ce6 were negligible because of the absence of AuDD. The time-dependent temperature increase in AuDD/BSA@CNf-Ce6 by NIR lasers was also visually validated at pH 7.4 and 6.8 (Figure 4b). Together, the results suggest that AuDD/BSA@CNf-Ce6 includes both photosensitizing Ce6 (Figure 3d) and the thermogenic AuNP, and they are activated at tumor-specific pH_e_ under light exposure, although the photothermal difference between pH 7.4 and 6.8 is not significantly different.

### 3.3. Cellular Entry Identification of AuDD/BSA@CNf-Ce6

**Figure 5:**
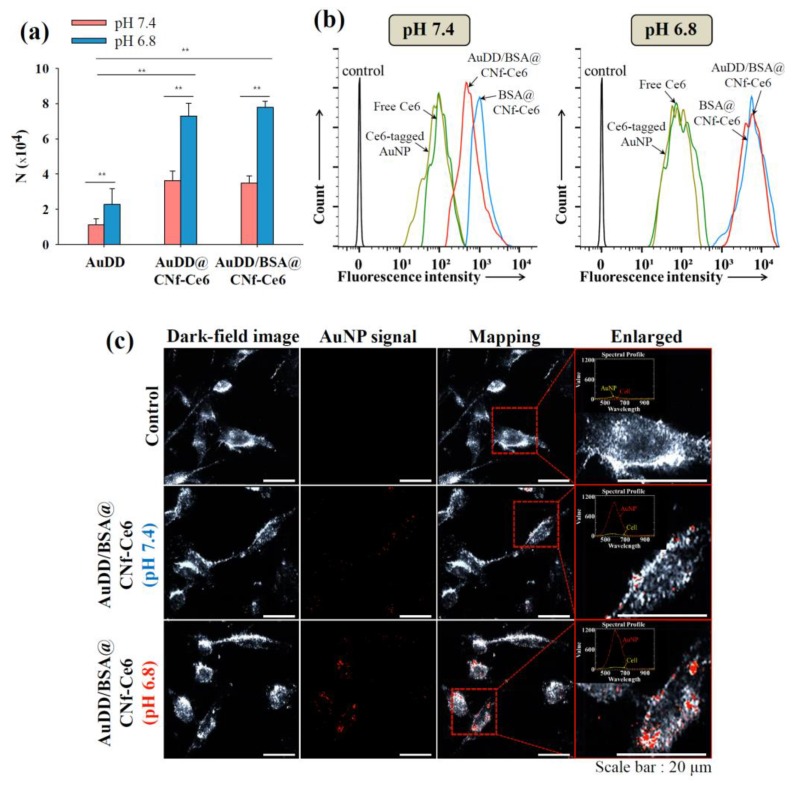
(**a**) Uptake value [number (N) of internalized AuDD particles per MDA-MB-231 cell for 8 h of incubation at 37 °C] of the AuDD-based samples measured using an ICP-MS (mean ± SD, n = 3, ** *p* < 0.01 compared to AuDD at pH 7.4). (**b**) Flow cytometry analysis of MDA-MB-231 cells (incubation time: 8 h) treated with free Ce6, Ce6-tagged AuNP, BSA@CNf-Ce6, or AuDD/BSA@CNf-Ce6 at pH 7.4 and 6.8. (**c**) The hyperspectral images of the MDA-MB-231 cells incubated with untreated (control) or AuDD/BSA@CNf-Ce6 at pH 7.4 and 6.8.

To evaluate the endocytic activity of AuDD/BSA@CNf-Ce6 in tumor cells, we treated such cells in the human breast adenocarcinoma cell line, MDA-MB-231, and measured the activity using the inductively coupled plasma mass spectrometry (ICP-MS) method [33] and flow cytometry. Figure 5a shows that the acidic pH of 6.8 significantly increased the quantitative cellular uptake of the AuDD-based nanostructures, suggesting that pH is essential in endocytosis. Notably, AuDD@CNf-Ce6 and AuDD/BSA@CNf-Ce6 at pH 6.8 exhibited the highest endocytic activity among the groups, as the positive charges of particles (Figure 3b) stimulate general clathrin-mediated endocytosis [46]. In addition, the filamentous structure of AuDD@CNf-Ce6 and AuDD/BSA@CNf-Ce6 improved endocytic activity at pH 7.4 and 6.8, when compared to non-filamentous AuDD [26,27]. In the flow cytometry analysis, Ce6-based fluorescence was observed, and the signal from AuDD/BSA@CNf-Ce6-treated cells at pH 6.8 was approximately 6.5-fold higher than at pH 7.4 (Figure 5b). In a qualitative manner, the signal of AuNP in AuDD/BSA@CNf-Ce6 was monitored at pH 7.4 and 6.8 using hyperspectral imaging analysis (Figure 5c).

**Figure 6:**
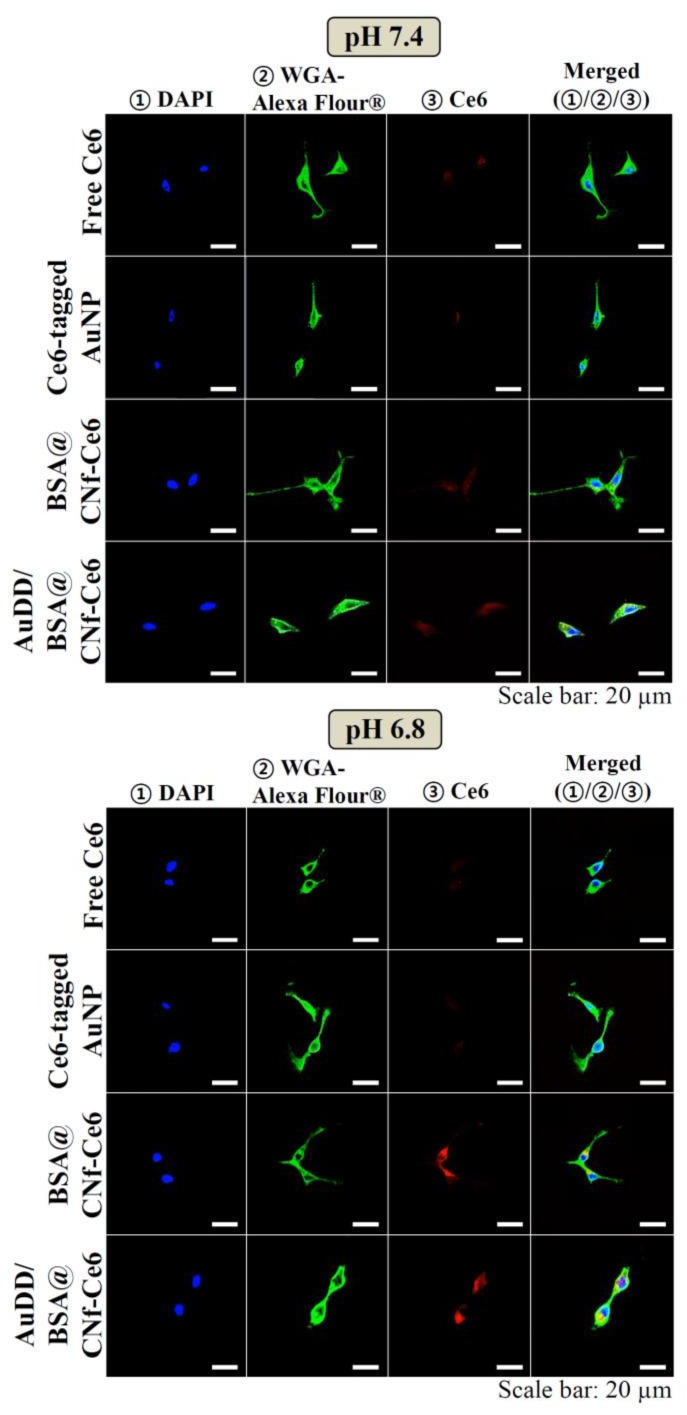
Confocal images of the MDA-MB-231 cells treated with free Ce6, Ce6-tagged AuNP, BSA@CNf-Ce6, or AuDD/BSA@CNf-Ce6 at pH 7.4 and 6.8. The cells were stained using DAPI and WGA-Alexa Fluor^®^488.

The images indicated that pH_e_-sensitive AuDD/BSA@CNf-Ce6 resulted in more AuNP uptake into cells at pH 6.8 by the stronger red signals. This is consistent with confocal images as shown in Figure 6. Whereas the intracellular fluorescence intensity of Ce6 was weak in free Ce6, Ce6-tagged AuNP, BSA@CNf-Ce6, and AuDD/BSA@CNf-Ce6 at pH 7.4, the red signal of Ce6 was remarkably increased in AuDD/BSA@CNf-Ce6 at pH 6.8, suggesting that AuDD/BSA@CNf-Ce6 has an effective intracellular delivery activity at pH_e_. The little red fluorescence from free Ce6 and Ce6-tagged AuNP was, however, detected because of their poor interaction with the cell membrane. Interestingly, BSA@CNf-Ce6 also showed strong red signals at pH 6.8, possibly because of its chitosan-based positive charge in acidic conditions and its filamentous shape [47].

### 3.4. In Vitro Phototoxicity Test

**Figure 7:**
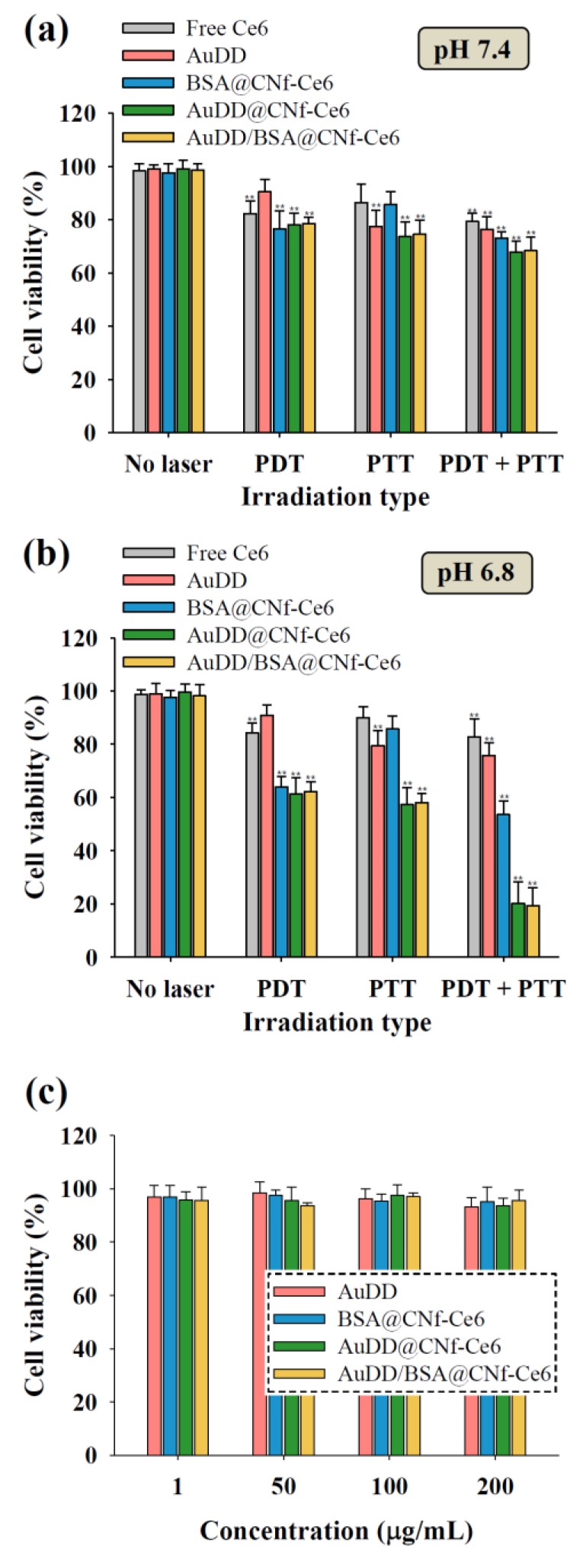
Cell viability of the MDA-MB-231 cells incubated with free Ce6 (10 μg/mL), AuDD (50 μg/mL), and CNf-Ce6-based samples (200 μg/mL, equivalent to Ce6 10 μg/mL) at (**a**) pH 7.4 and (**b**) pH 6.8 (mean ± SD, *n* = 8, ** *p* < 0.01 compared to no laser). The cells were irradiated for 10 min at a light intensity of 5.2 mW/cm^2^ using a 670 nm laser source and/or for 5 min at a light intensity of 2 W/cm^2^ using an 808 nm laser source. (**c**) Cell viability of the MDA-MB-231 cells incubated with AuDD or CNf-Ce6-based samples for 24 h at 37 °C without light irradiation (mean ± SD, *n* = 8, ** *p* < 0.01 compared to AuDD).

To explore the photothermal and photodynamic activities of AuDD/BSA@CNf-Ce6 against tumor cells, MDA-MB-231 cells were treated with the nanostructure at pH 6.8 and 7.4, followed by irradiation with 670 nm visible light for singlet oxygen generation (PDT) and/or an 808 nm NIR laser for heat production (PTT) (Figure 7). The tumor cell death by combined phototherapy at pH 7.4 showed changes that were not significant when compared to death by mono-phototherapy (Figure 7a). However, AuDD@CNf-Ce6 and AuDD/BSA@CNf-Ce6 dramatically reduced the tumor cell viability by dual light irradiation at pH 6.8 (Figure 7b), suggesting that they exhibit a synergistic effect for tumor cell inactivation at pH_e_ in vitro. This is because pH_e_ leads to the active division of AuDD and CNf-Ce6, and the wavelengths of 670 and 808 nm activate photodynamic Ce6 and photothermic AuNP from CNf-Ce6 and AuDD, respectively. Interestingly, combined phototherapy resulted in higher antitumor efficacy than the repeated single therapy of PDT or PTT at pH 6.8 in MDA-MB-231 cells and human glioblastoma T98G cells (Figure S5). Moreover, the therapeutic order did not affect the phototoxicity in both tumor cell lines, a differing result from the previous order-dependent phototherapy reports [36,41]. The pH_e_-triggered AuDD detachment from AuDD/BSA@CNf-Ce6 is responsible for the dequenching event of Ce6, which allows the order-independent dual-phototherapy. To determine nanostructure-induced cytotoxicity, the cell integrity was measured for a wide range of material concentrations (Figure 7c). The results consistently presented high cell viability, regardless of concentration, indicating that the nanostructures are non-cytotoxic.

**Figure 8:**
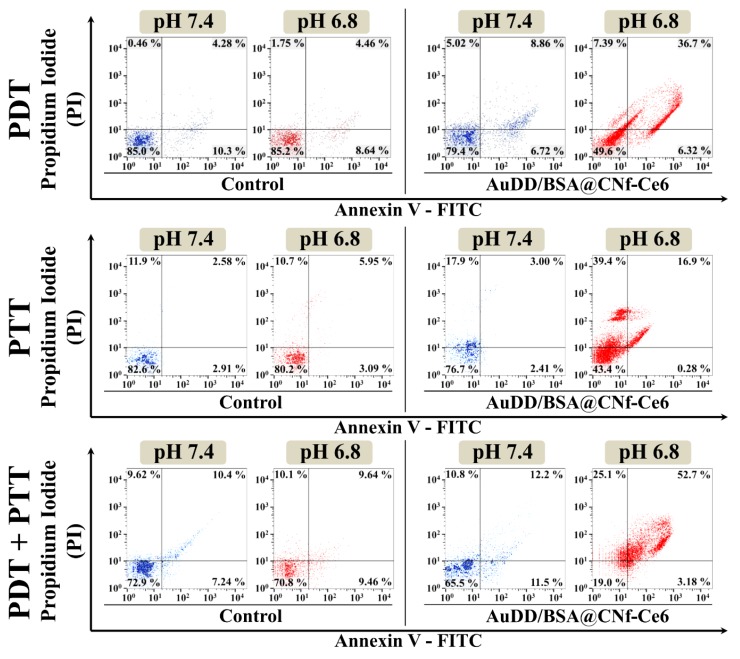
Flow cytometry results of apoptosis/necrosis using control (untreated) or AuDD/BSA@CNf-Ce6 (200 μg/mL, equivalent to Ce6 10 μg/mL) under PDT (670 nm, 5.2 mW/cm^2^) treatment, PTT (808 nm, 2 W/cm^2^) treatment, or combined PDT/PTT treatment. The results of apoptosis/necrosis were determined using Annexin V-FITC and PI staining. Each quadrant is indicated as follows: lower left, live cells; lower right, early apoptotic cells; upper left, dead cells; upper right, late apoptotic/necrotic cells.

Apoptotic and/or necrotic tumor cell death was evaluated by flow cytometry using the special dyes, Annexin V-fluorescein isothiocyanate (FITC) and propidium iodide (PI), for early apoptotic and late apoptotic/necrotic cells, respectively [37]. We found that the different types of phototherapy and pH did not affect cell death in the untreated control groups (Figure 8, lower left quadrants in control groups). Under physiological conditions of pH 7.4, PDT, PTT, and the combination of PDT and PTT with AuDD/BSA@CNf-Ce6 did not result in a prominent increase in late apoptosis/necrosis when compared to their corresponding control. However, the AuDD/BSA@CNf-Ce6 treatment followed by the phototherapies strikingly augmented cell death at pH 6.8 (Figure 8, upper right quadrants in AuDD/BSA@CNf-Ce6 groups). This indicated that the pH_e_ ignited Ce6-induced PDT and/or AuNP-triggered PTT, resulting in potent cell death. In particular, the mixture of PDT and PTT showed the highest rates of apoptosis and necrosis, 52.7%, which differed from individual PDT of 36.7% and PTT of 16.9%. This suggested that combined phototherapy with AuDD/BSA@CNf-Ce6 enhance phototoxicity against tumor cells in response to pH_e_.

### 3.5. In Vivo Antitumor Study

**Figure 9:**
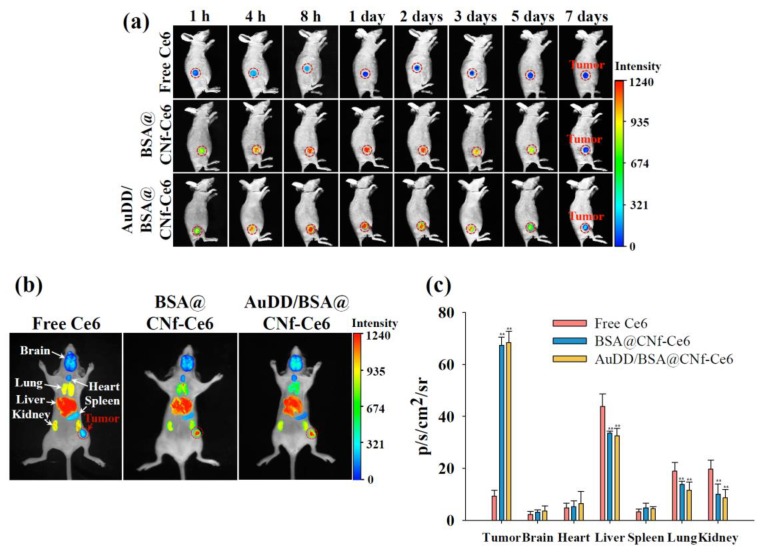
(**a**) In vivo non-invasive photoluminescent tumor images of free Ce6, BSA@CNf-Ce6, or AuDD/BSA@CNf-Ce6 injected intravenously into MDA-MB-231 tumor-bearing nude mice. Fluorescent tumor images were obtained for 7 d post-injection. Tumor sites are indicated by the dashed circles. (**b**) Fluorescence images of free Ce6, BSA@CNf-Ce6, or AuDD/BSA@CNf-Ce6 into organs harvested from MDA-MB-231 tumor-bearing nude mice at 8 h post-injection. (**c**) Total photon counts per centimeter squared per steradian (p/s/cm^2^/sr; measured using an Image Station 4000 MM) of the organs harvested from MDA-MB-231 tumor-bearing nude mice at 8 h post-injection (mean ± SD, n = 5, ** *p* < 0.01 compared to free Ce6).

**Figure 10:**
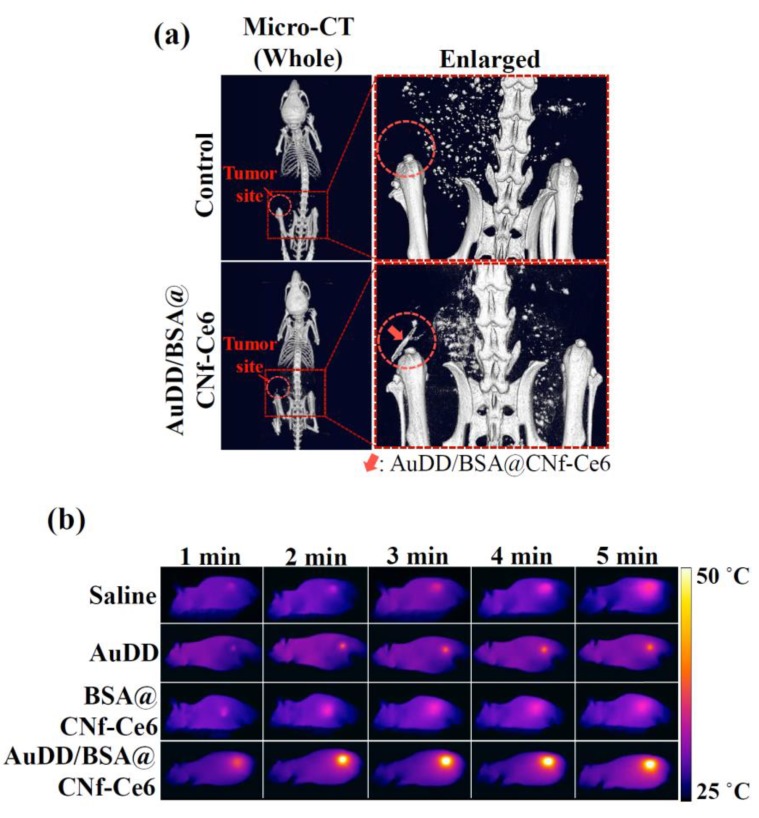
(**a**) In vivo micro-CT images of MDA-MB-231 tumor-bearing nude mice treated with saline (control) or AuDD/BSA@CNf-Ce6 at 8 h post-injection. The tumor sites are indicated by the dashed circles. (**b**) In vivo whole-body thermo-graphic images of the MDA-MB-231 tumor-bearing nude mice treated with saline (control), AuDD, BSA@CNf-Ce6, or AuDD/BSA@CNf-Ce6. The tumor sites were irradiated at a light intensity of 2 W/cm^2^ using an 808 nm light source for 5 min after the 8 h administration.

Encouraged by the results of the in vitro tumor inhibition activity by AuDD/BSA@CNf-Ce6, animal experiments were performed. First, to assess tumor-targeting efficiency, the free Ce6, BSA@CNf-Ce6, and AuDD/BSA@CNf-Ce6 were administered to MDA-MB-231 tumor-bearing nude mice. Figure 9a demonstrates that the free Ce6 had negligibly detectable signals in the tumor region during the entire phase. In contrast, both BSA@CNf-Ce6 and AuDD/BSA@CNf-Ce6 produced visible fluorescence signals until the late phase (5 d after application). To observe the delivery point in depth, isolated organs from the nanostructure-treated mice were visualized (Figure 9b). The ex vivo images demonstrated that free Ce6 was mainly delivered to the liver, a marked contrast to the tumor-tropic BSA@CNf-Ce6 and AuDD/BSA@CNf-Ce6. To quantify the fluorescence, total photon was measured as 9.4, 67.4, and 68.4 photons of free Ce6, BSA@CNf-Ce6, and AuDD/BSA@CNf-Ce6, respectively, found in tumors (Figure 9c). Both BSA@CNf-Ce6 and AuDD/BSA@CNf-Ce6 have BSA for stability in blood [30], and a fiber structure for durability in the body [26,27]; these characteristics led to the strong and long-lasting detection in tumor cells (Figure 9a–c). Importantly, the measured photons in BSA@CNf-Ce6 and AuDD/BSA@CNf-Ce6 were significantly reduced in the liver, lung, and kidney, suggesting that the DDS tools inhibit non-specific distribution of payloads (Figure 9c). A micro-computed tomography (micro-CT) assay confirmed tumor-selective AuDD/BSA@CNf-Ce6 delivery with high definition (Figure 10a). To validate the heat generation of AuDD/BSA@CNf-Ce6, whole-body of mice was graphically monitored for 5 min (Figure 10b). AuDD/BSA@CNf-Ce6 reached approximately 50 °C in the tumor region after 2 min of irradiation owing to its tumor-tropic distribution. However, the saline, AuDD, and BSA@CNf-Ce6 groups displayed negligible signals (Figure 10b). Even though BSA@CNf-Ce6 had tumor-headed movement (Figure 9a–c), the AuDD-deficiency revoked its photothermal activity at the tumor site.

**Figure 11:**
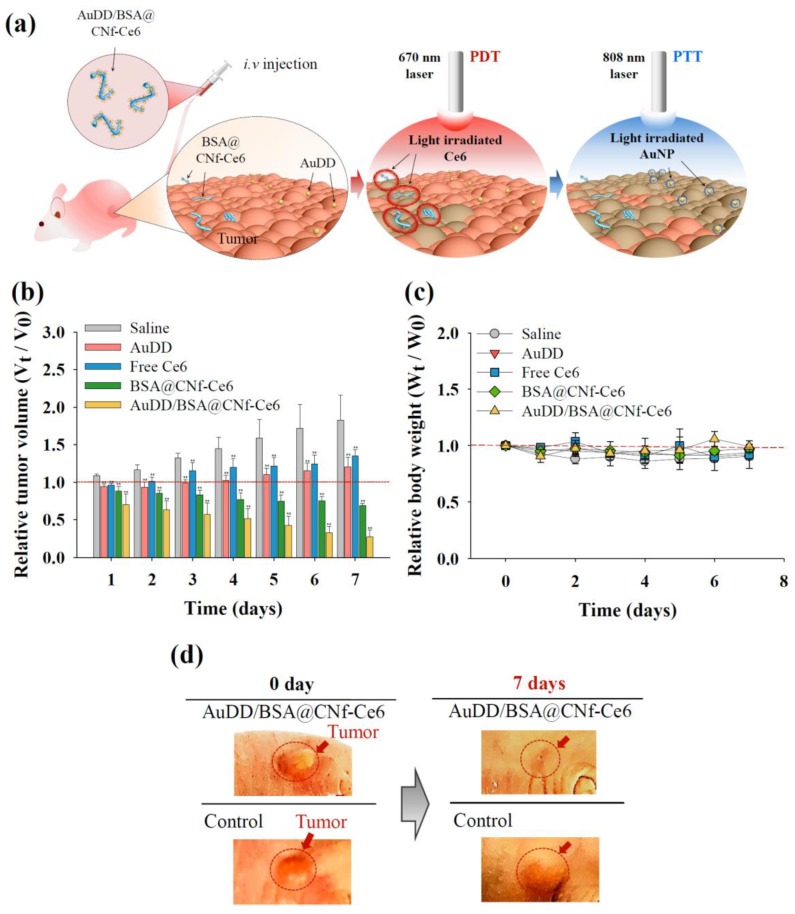
(**a**) Graphical strategy of AuDD/BSA@CNf-Ce6 for PDT/PTT dual therapy. (**b**) Relative tumor volume change (*V_t_*/*V*_0_) and (**c**) relative body weight change (*W_t_*/*W*_0_) of MDA-MB-231 tumor-bearing nude mice treated with saline (control), AuDD, free Ce6, BSA@CNf-Ce6, or AuDD/BSA@CNf-Ce6. Tumor sites were irradiated at a light intensity of 5.2 mW/cm^2^ for 40 min using a 670 nm laser source at 8 h post-injection (PDT treatment), then at a light intensity of 2 W/cm^2^ using an 808 nm light source for 5 min (PTT treatment) (mean ± SD, *n* = 5, ** *p* < 0.01 compared to saline). (**d**) Optical photographs of MDA-MB-231 tumor-bearing nude mice after 7 d of injection. Tumor sites are indicated by the dashed circles.

To investigate the efficacy and safety of the antitumor devices, we divided mice into the following five treatment groups: (1) saline, (2) AuDD, (3) free Ce6, (4) BSA@CNf-Ce6, and (5) AuDD/BSA@CNf-Ce6. Each group was treated with 670 nm laser irradiation for PDT, and an additional 808 nm laser irradiation for PTT (Figure 11a–c). After the single injection of each material into the equivalent group, tumor size was measured (Figure 11b). AuDD/BSA@CNf-Ce6 elicited the strongest tumor growth inhibition among the groups (Figure 11b,d), a result of pH_e_-sensitivity (Figure 3), charge and shape-stimulated endocytosis (Figure 5 and Figure 6), and tumor tropism (Figure 9 and Figure 10). Free Ce6 and AuDD exhibited mild effects on tumor inhibition because of their poor tumor accumulation, while BSA@CNf-Ce6 demonstrated acceptable antitumor activity because of efficient tumor distribution (Figure 9). However, BSA@CNf-Ce6 displayed larger tumor volume than AuDD/BSA@CNf-Ce6 (Figure 10b). This indicated that AuDD/BSA@CNf-Ce6 administration results in the most effective and synergistic tumor cell death attributable to its two-way phototherapy. Markedly, the tumor regression caused by AuDD/BSA@CNf-Ce6 continued after a week of single-dosed administration (Figure 11b), suggesting that this therapeutic approach is viable, sustainable, and patient-friendly. Changes in body weight were negligible among the five treatment groups (Figure 11c), a result that aligns with the in vitro cytotoxicity test results (Figure 7c). Overall, the data have reinforced the tumor-killing efficacy and safety of dual PDT/PTT-intended AuDD/BSA@CNf-Ce6 treatment in vivo.

## 4. Conclusions

Herein, AuDD/BSA@CNf-Ce6 was strategically designed and developed to selectively release phototoxins against tumors. Its backbone was composed of worm-like, biocompatible CNf, covalently conjugated to the photodynamic Ce6. In addition, BSA and heat-generating AuNP were electrostatically linked to the chitosan body to improve the blood circulation of the carrier. The interaction between AuDD/BSA@CNf-Ce6 and tumor-specific pH_e_ initiated the separation and launch of CNf-Ce6 and AuDD, which synergistically induced tumor damage upon exposure to different wavelengths of radiation. Collectively, pH_e_-sensitive AuDD/BSA@CNf-Ce6 engineered for combined PDT and PTT might unlock the maximum therapeutic potential against tumor, thus functioning as a promising next-generation DDS tool. We will conduct a detailed in vivo study and a pharmacokinetic study in the future.

## Figures and Tables

**Table 1:** Weight fractions of each component in the CNf-Ce6-based nanofibers.

Sample	Weight Fraction (%)		
	AuDD	Ce6	BSA
BSA@CNf-Ce6	-	5.1	4.1
AuDD@CNf-Ce6	24.9	4.9	-
AuDD/BSA@CNf-Ce6	25.5	5.2	3.9

## Supplementary Materials

The following are available online at https://www.mdpi.com/1999-4923/11/6/258/s1, Figure S1: ^1^H-NMR spectrum of dopamine-DMMA (300 MHz, CDCl3). Figure S2: Particle size distribution and TEM image of AuDD. Figure S3: Hyperspectral images of AuDD, CNf, and the different types of AuDD@CNf. The linked amount (%) of each AuDD to CNf is 7% (AuDD7@CNf), 24% (AuDD24@CNf) and 40% (AuDD40@CNf). Figure S4: Binding efficiency of AuDD to CNf-Ce6 with different feeding amounts. The optimal feeding ratio of AuDD for AuDD@CNf-Ce6 is indicated by the dashed rectangle. Figure S5: Cell viability determined by the CCK-8 assay of (a) MDA-MB-231 cells and (b) T98G cells incubated with AuDD/BSA@CNf-Ce6 (200 μg/mL, equivalent to Ce6 10 μg/mL) at pH 6.8 (mean ± SD, *n* = 8, ** p < 0.01 compared to dual-PDT irradiation). The cells were irradiated for 10 min at a light intensity of 5.2 mW/cm2 using a 670-nm laser source for PDT and/or for 5 min at a light intensity of 2 W/cm2 using an 808-nm laser source for PTT. Table S1: Degree of deacetylation and amine concentration of chitosan nanofiber (CNf) obtained by Fourier transform infrared spectroscopy and titration, *n* = 3, mean ± standard deviation).
