# Supplementary material for: Poisonous Caterpillar-Inspired Chitosan Nanofiber Enabling Dual Photothermal and Photodynamic Tumor Ablation
Source: Pharmaceutics. 2019 Jun 2;11(6):258. doi: 10.3390/pharmaceutics11060258 (PMC6631857; doi:10.3390/pharmaceutics11060258)
Supplement: Supplementary file 1 [file pharmaceutics-11-00258-s001.pdf]

pharmaceutics

Supplementary Materials: Poisonous Caterpillar-Inspired Chitosan Nanofiber Enabling Dual Photothermal and Photodynamic Tumor Ablation

Hyeong Sup Yu, Hongsuk Park, Thang Hong Tran, Sung Yeon Hwang, Kun Na, Eun Seong Lee, Kyung Taek Oh, Dongyeop X. Oh, and Jeyoung Park

Table S1. Degree of deacetylation and amine concentration of chitosan nanofiber (CNf) obtained by Fourier transform infrared spectroscopy and titration (n = 3, mean ± standard deviation).

	Degree of deacetylation (%)	
	FT-IR	Titration
CNf	44.0	26.9 ± 3.3

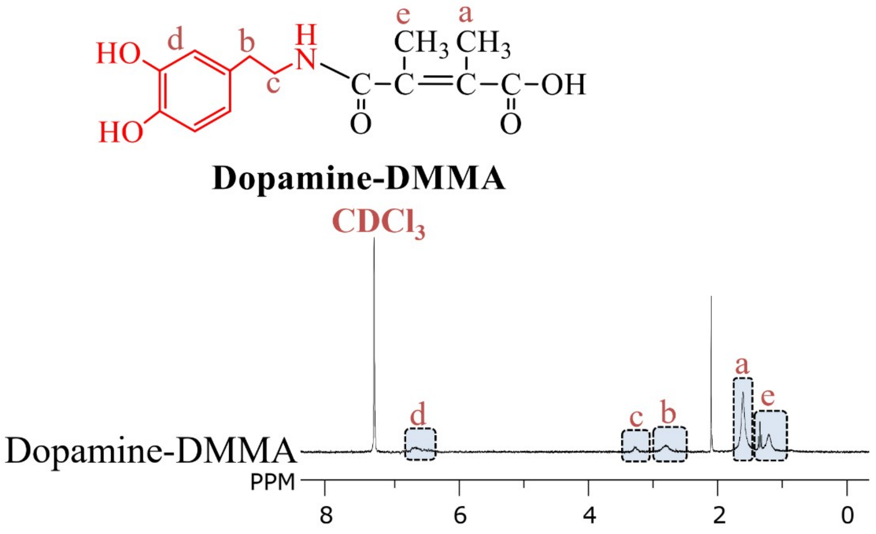

Figure S1. ^{1}H-NMR spectrum of dopamine-DMMA (300 MHz, $CDCl_3$).

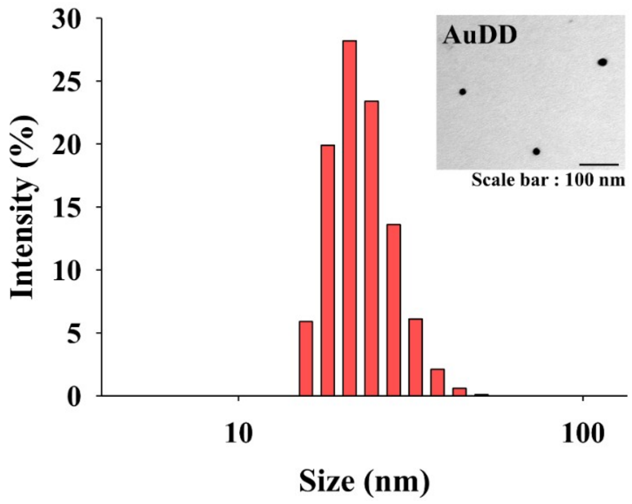

Figure S2. Particle size distribution and TEM image of AuDD.

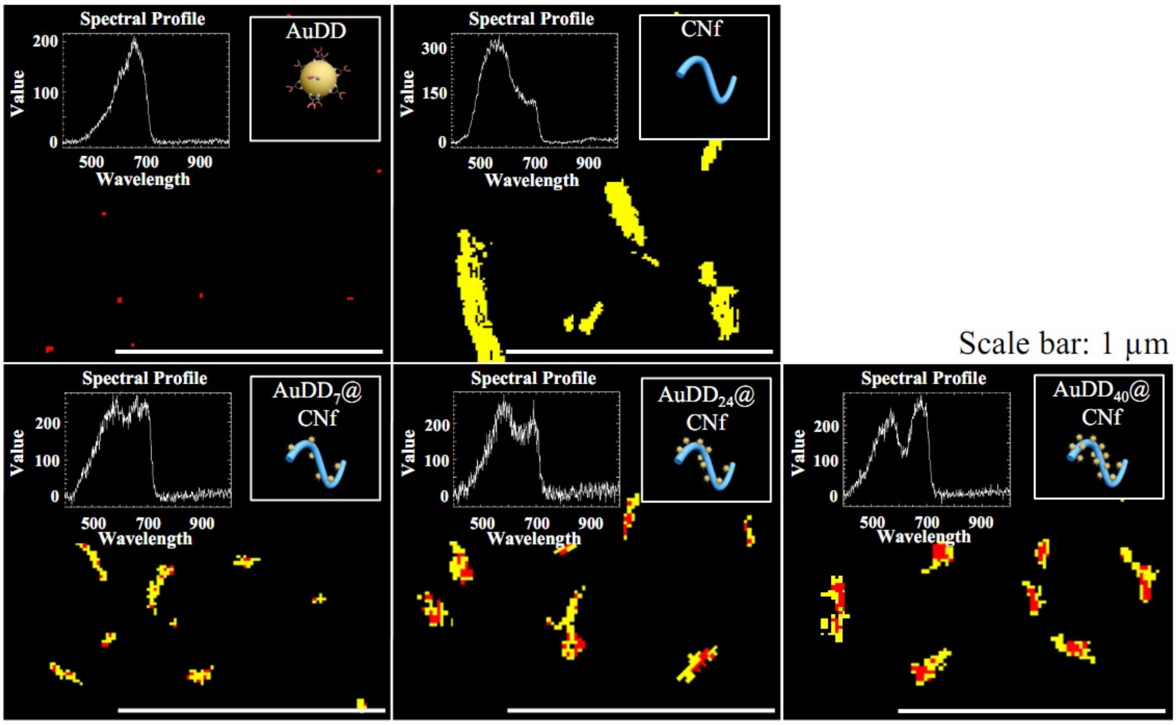

Figure S3. Hyperspectral images of AuDD, CNf, and the different types of AuDD@CNf. The linked amount (%) of each AuDD to CNf is 7% (AuDD$_7$@CNf), 24% (AuDD$_{24}$@CNf) and 40% (AuDD$_{40}$@CNf).

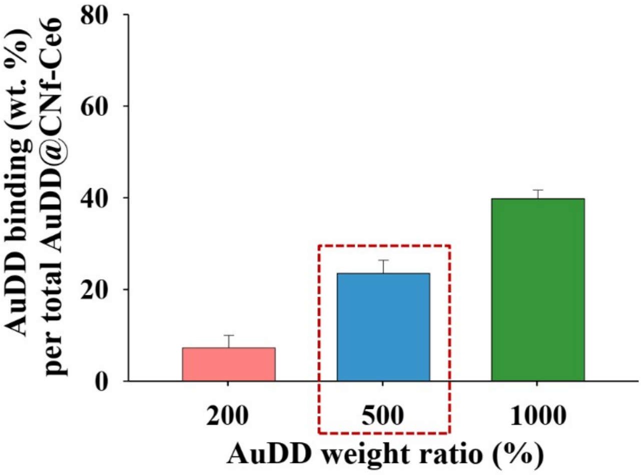

Figure S4. Binding efficiency of AuDD to CNf-Ce6 with different feeding amounts. The optimal feeding ratio of AuDD for AuDD@CNf-Ce6 is indicated by the dashed rectangle.

(a)

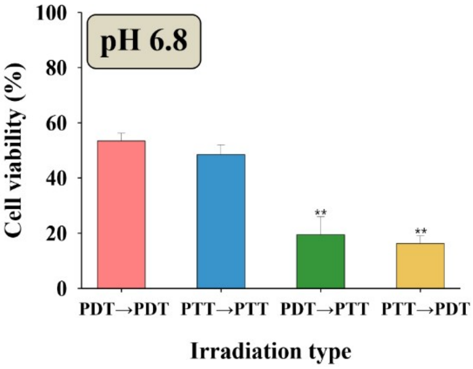

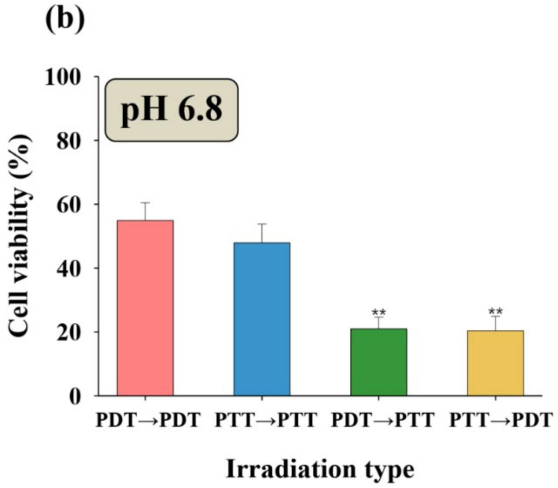

Figure S5. Cell viability determined by the CCK-8 assay of (**a**) MDA-MB-231 cells and (**b**) T98G cells incubated with AuDD/BSA@CNf-Ce6 (200 μg/mL, equivalent to Ce6 10 μg/mL) at pH 6.8 (mean ± SD, n = 8, ** $p < 0.01$ compared to dual-PDT irradiation). The cells were irradiated for 10 min at a light intensity of 5.2 mW/cm^2 using a 670-nm laser source for PDT and/or for 5 min at a light intensity of 2 W/cm^2 using an 808-nm laser source for PTT.
